# DFT calculation and NMR data of novel aryloxymaleimides and the intermediates and transition states in the reaction

**DOI:** 10.1016/j.dib.2019.104110

**Published:** 2019-06-08

**Authors:** Maocai Yan, Zhen Zhang, Jinhui Zhou, Wei Li, Chunyan Zhang, Shuai Fan, Zhaoyong Yang

**Affiliations:** a School of Pharmacy, Jining Medical University, Rizhao, Shandong, 276800, China; b Institute of Medicinal Biotechnology, Chinese Academy of Medical Sciences and Peking Union Medical College, Beijing, 100050, China

**Keywords:** Aryloxymaleimides, Transition states, Intermediates, NMR data, Density functional theory

## Abstract

Maleimide ring is an important scaffold in organic chemistry, and tosyloxy group is a functional group widely used in organic synthesis. Nevertheless, tosyloxymaleimide compounds have been rarely reported, and the reactivity properties and potential applications of tosyloxymaleimide in organic synthesis remain to be explored. This article presents the density functional theory (DFT) calculation data of the reaction mechanism of nucleophilic substitutions of tosyloxymaleimide with phenol, including the coordinate of all the stationary points (the reactant, transition states, intermediates, and product). All the structures had been geometrically optimized using M06-2X functional and 6-31+G** basis set; the reactant, intermediates and product had no imaginary frequencies, and each transition state has only one imaginary frequency in the vibration analysis at the same computation level. The intrinsic reaction coordinates (IRCs) of two steps of the reaction were calculated. ^1^H and ^13^C NMR spectra of the novel aryloxymaleimide compounds synthesized using this nucleophilic substitution reaction (doi: 10.1016/j.molstruc.2019.04.020 Yan et al.,) were also presented in this article.

**Table:** Specifications table

Subject area	*Chemistry*
More specific subject area	*Theoretical chemistry, organic chemistry, reaction mechanism*
Type of data	*Coordinate, Figure*
How data was acquired	*DFT calculation (Gaussian 09, Gaussian Inc.), NMR (*Bruker Avance III-400 and Ascend™ 600 spectrometers*)*
Data format	*Raw, analyzed*
Experimental factors	*Synthesized samples were dissolved in CDCl*_*3*_*or DMSO-d*_*6*_*before NMR determination*
Experimental features	*DFT calculations at M06-2X/6-31*+G** level; NMR spectra detected on Bruker Avance III-400 or Ascend^*TM*^*600 spectrometers*
Data source location	*School of Pharmacy, Jining Medical University, Rizhao, Shandong, 276800, China*
Data accessibility	*Data is with this article*
Related research article	*Yan, M.; Zhang, Z.; Zhou, J.; Li, W.; Zhang, C.; Zhang, J.; Wang, H.; Yang, X.; Fan, S.; Yang, Z. Synthesis and DFT studies of novel aryloxymaleimides via nucleophilic substitution of tosyloxy group. Journal of Molecular Structure, 1189 (2019), 155–160.**[1]*

**Table:** 

**Value of the data** • This article presents geometrically optimized structures and coordinates of the transition states, intermediates, reactant and product of a representative nucleophilic substitution reaction (tosyloxymaleimide reacting with phenoxy anion to give phenoxymaleimide); these coordinates would be useful to researchers who are interested in the modeling of this system or similar systems. • The intrinsic reaction coordinates of the two elementary reactions were calculated; these may provide more details and clues for the reaction mechanisms. • ^1^H and ^13^C NMR spectra of ten novel aryloxymaleimides were provided, which are useful for structure characterization of aryloxymaleimide compounds.

## 1 Data

**Fig. 1:**
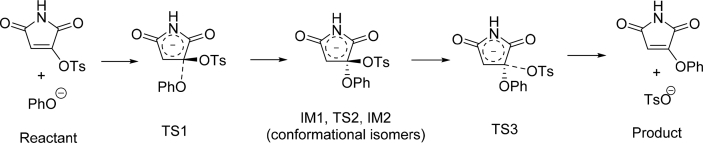
The reaction process of nucleophilic substitution of tosyloxy with phenoxy anion. “TS” and “IM” indicate transition state and intermediate, respectively.

**Fig. 2:**
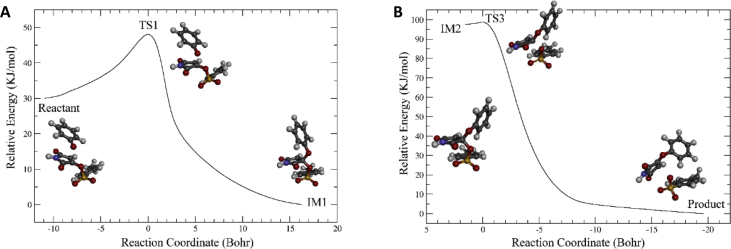
(A) IRC of the nucleophilic addition reaction of 3 with phenoxy anion; (B) IRC of elimination of tosyloxy anion from the tetrahedral intermediate.

Maleimide is a common substructure in organic compounds, and tosyloxy (TsO^−^) group is an important functional group widely used in organic synthesis because it is an excellent leaving group. However, surprisingly, compounds with a tosyloxy group on maleimide ring have been very rarely reported [2]. [3] In the related research article [1], we synthesized ten novel aryloxymaleimide compounds by reacting tosyloxymaleimide with various phenols. Here we present the density functional theory (DFT) calculation data for the mechanism of this nucleophilic substitution reaction, as well as the ^1^H and ^13^C NMR spectra of the newly synthesized compounds. The three-dimensional Cartesian coordinates of all the stationary points of the reaction process, after geometry optimization using DFT (M06-2X functional and basis set 6-31+G**), are listed as plain texts. Fig. 1 illustrates the whole reaction process; the Gibbs free energy profile and the conformational difference among IM1, TS2, and IM2 were given in the related research [1]. Intrinsic reaction coordinates (IRCs) of the two elementary reactions, nucleophilic addition of the phenoxy anion and elimination of the tosyloxy anion, are shown in Fig. 2. The ^1^H and ^13^C NMR spectra of the newly synthesized aryloxymaleimide compounds and the reactant tosyloxymaleimide are available in the Supplementary Information.

## 2 Experimental design, materials, and methods

The chemical synthesis of aryloxymaleimide compounds and the starting material tosyloxymaleimide have been reported in our previous work [1] [4] . ^1^H and ^13^C NMR spectra were detected on Bruker Avance III-400 or Ascend™ 600 spectrometers. The samples were dissolved in CDCl_3_ or DMSO-*d*_6_ before NMR determination.

The DFT calculations were done with Gaussian 09 [5]. The Solvation Model based on Density (SMD) implicit solvation model [6] of CH_2_Cl_2_ (the actual solvent of the reaction) was used in all DFT calculations. The hybrid-meta GGA functional M06-2X [7], [8], in combination of the basis set 6-31+G** [9], [10], was used in all the theoretical calculations presented in this article, including geometry optimization, vibration analysis, and calculation of IRCs. All the stationary point structures have been subject to geometry optimization and then vibration analysis; each transition state has only one imaginary frequency, and the intermediates, the reactant, and the product have no imaginary frequencies. In IRC calculation, local quadratic approximation (LQA) method [11], [12] was used and the step size was set to 0.05 Bohr, and the Hessian matrix was recalculated every 5 steps; a maximum steps of 300 was set in each direction, and all IRC calculations finished within 300 steps.

**Table:** 

Coordinates of Reactant:			
C	0.41460300	−0.93099200	0.09949200
C	0.42983700	0.28732200	0.65048900
H	−0.17668500	1.15532600	0.42989900
N	2.08119400	−0.98795300	1.65046100
O	−0.38683400	−1.42349500	−0.89274300
C	1.52035800	0.28526500	1.66705300
C	1.44188700	−1.81855500	0.74246200
O	1.61353000	−3.00719900	0.59575100
O	1.85050700	1.18799100	2.41078700
H	2.85932800	−1.27090200	2.23378700
S	−1.91949000	−1.86310100	−0.48112500
O	−2.40839500	−2.48742500	−1.69252700
O	−1.85972700	−2.59578100	0.76939300
C	−2.71977900	−0.31424900	−0.21643800
C	−3.00345300	0.49090100	−1.32014600
C	−3.00533500	0.08774400	1.08484400
C	−3.59351800	1.72885900	−1.10096200
H	−2.76499400	0.15751800	−2.32595000
C	−3.60115400	1.33116800	1.27963600
H	−2.76219900	−0.55580700	1.92427500
C	−3.90240000	2.16440200	0.19662500
H	−3.82055500	2.36947800	−1.94876900
H	−3.82996800	1.65958500	2.28956500
C	−4.57039000	3.49511700	0.41030100
H	−5.64756900	3.40989700	0.22801900
H	−4.42903600	3.84902800	1.43404500
H	−4.17722700	4.24609700	−0.28002700
O	2.52852000	−0.85191400	−1.42375600
C	3.04926600	0.27198900	−1.09090000
C	4.30179800	0.36255500	−0.39622100
C	2.40285600	1.52358200	−1.35421300
C	4.84557400	1.58100300	−0.01546600
H	4.82015000	−0.56809500	−0.17279100
C	2.96332400	2.73888100	−0.96676200
H	1.45703800	1.49760700	−1.89180500
C	4.18462700	2.78847200	−0.28879200
H	5.79966200	1.59839300	0.50850800
H	2.43608400	3.66385900	−1.19387500
H	4.61437100	3.73801500	0.01575800
Coordinates of TS1:			
C	0.63593900	−0.90618600	0.01004400
C	0.46091200	0.21190900	0.78682000
H	−0.12798400	1.09254400	0.57723200
N	2.00749700	−1.16346900	1.81051300
O	−0.25045100	−1.39020700	−0.94421200
C	1.38335600	0.10641400	1.89955700
C	1.49159600	−1.89863500	0.77043800
O	1.67808200	−3.07286300	0.53184000
O	1.62972400	0.89328800	2.80407300
H	2.59973700	−1.54385100	2.53863600
S	−1.72472300	−1.86922700	−0.44135400
O	−2.25133600	−2.55851900	−1.60466000
O	−1.59839600	−2.56077100	0.82900800
C	−2.60300000	−0.35369200	−0.20431900
C	−2.89901200	0.42591700	−1.32213400
C	−2.95891800	0.03764500	1.08205000
C	−3.56796500	1.62898800	−1.13423900
H	−2.60724600	0.10110900	−2.31666200
C	−3.63458100	1.24414600	1.24667300
H	−2.70216000	−0.58314800	1.93447100
C	−3.94593100	2.05329800	0.14809300
H	−3.80200400	2.25015900	−1.99458600
H	−3.91629400	1.56501200	2.24571000
C	−4.69436200	3.34587600	0.32995700
H	−5.76318600	3.19488800	0.14128300
H	−4.58354900	3.72715300	1.34778300
H	−4.33934000	4.10617400	−0.37083900
O	2.03586800	−0.72442900	−1.32257400
C	2.79955200	0.31637900	−1.06787200
C	4.12590300	0.15327700	−0.59772300
C	2.31958700	1.63840200	−1.21828800
C	4.93472700	1.25240900	−0.33266400
H	4.49803700	−0.85951600	−0.45967300
C	3.13487500	2.73540200	−0.93987300
H	1.30503600	1.77930300	−1.58171700
C	4.44584900	2.55425100	−0.49728400
H	5.95392300	1.09729300	0.01342200
H	2.74148600	3.74067200	−1.07079500
H	5.07846900	3.41042400	−0.28255900
Coordinates of IM1:			
C	0.86809900	−0.46652200	−0.29329300
C	0.47171400	0.57969800	0.63460700
H	−0.04151400	1.49207300	0.36658900
N	1.53110000	−1.05344100	1.85928700
O	−0.19629900	−1.11886300	−1.09119200
C	0.83646800	0.22305400	1.93181200
C	1.53969800	−1.55338400	0.60920500
O	1.99493500	−2.61161400	0.20946900
O	0.68807800	0.76003700	3.04215800
H	1.81664900	−1.56931200	2.68234100
S	−1.52354500	−1.66280500	−0.38226100
O	−2.13535000	−2.50155400	−1.40244600
O	−1.19475400	−2.23432900	0.91487800
C	−2.55253500	−0.23734800	−0.14957000
C	−3.07375600	0.39914900	−1.27403600
C	−2.83037800	0.20690600	1.13844100
C	−3.88734300	1.51238400	−1.09371900
H	−2.84550000	0.03431500	−2.27139600
C	−3.65434200	1.31767900	1.29809000
H	−2.39624400	−0.29868200	1.99532700
C	−4.19226500	1.98334400	0.19055700
H	−4.29614300	2.02197700	−1.96235900
H	−3.87484600	1.67774400	2.29934700
C	−5.10488100	3.16650600	0.37134500
H	−6.15268000	2.84955700	0.31954600
H	−4.94715700	3.64210000	1.34244700
H	−4.94594200	3.91086300	−0.41355300
O	1.71180300	−0.13230000	−1.40023900
C	2.81698700	0.64004900	−1.10754900
C	3.96792800	0.05324000	−0.58243100
C	2.78664900	1.99974600	−1.41208500
C	5.09382600	0.84399600	−0.35159600
H	3.97700500	−1.01155500	−0.36977500
C	3.91913100	2.78031300	−1.18659200
H	1.87682800	2.42748900	−1.82227100
C	5.07366300	2.20608500	−0.65180300
H	5.99126900	0.39005700	0.05855100
H	3.89729100	3.83967700	−1.42516300
H	5.95370000	2.81629500	−0.47201900
Coordinates of TS2:			
C	0.99374300	−0.86430500	−0.23506500
C	0.57442200	−0.01196600	0.86392600
H	0.22733500	1.00710200	0.77276900
N	1.21608800	−2.02651400	1.76675200
O	−0.01029800	−1.12423200	−1.31187100
C	0.67314200	−0.71250500	2.06509600
C	1.35891000	−2.22324100	0.43911100
O	1.73270500	−3.22196300	−0.14896200
O	0.40705100	−0.42624700	3.24465400
H	1.28103800	−2.76156800	2.45981700
S	−1.48260500	−1.61997400	−0.93933800
O	−2.04127300	−2.03854700	−2.21715900
O	−1.42417100	−2.56970400	0.16262000
C	−2.34832100	−0.16718600	−0.40716200
C	−2.58736000	0.84225700	−1.33466900
C	−2.78107900	−0.06781000	0.91218700
C	−3.26730600	1.98468400	−0.92021700
H	−2.24746300	0.74170600	−2.36152500
C	−3.46806400	1.07612300	1.30377800
H	−2.57016900	−0.86600400	1.61690400
C	−3.71502900	2.11777200	0.39919200
H	−3.45650700	2.78150500	−1.63432500
H	−3.81145000	1.16678600	2.33106900
C	−4.43612600	3.35958600	0.85115700
H	−4.72881500	3.97915300	0.00019100
H	−5.33387400	3.10469700	1.42200800
H	−3.79213600	3.95967900	1.50309800
O	2.07776000	−0.47502200	−1.07664000
C	2.80264500	0.64093400	−0.72066600
C	3.84065000	0.52550500	0.20233600
C	2.52808500	1.85813700	−1.33876300
C	4.60763600	1.64698600	0.51380700
H	4.03221900	−0.43731900	0.66836600
C	3.30245000	2.97552800	−1.02375100
H	1.71184600	1.91717900	−2.05312600
C	4.34109600	2.87349700	−0.09776000
H	5.41390500	1.56146900	1.23646300
H	3.09183100	3.92722500	−1.50308700
H	4.94015200	3.74532200	0.14717000
Coordinates of IM2:			
C	1.33906000	−0.54221600	0.36649500
C	0.27172100	−0.58054700	1.35799200
H	−0.43869900	0.20287400	1.57023500
N	1.34377500	−2.61589500	1.41912700
O	0.94085900	−0.30012100	−1.07139000
C	0.24768100	−1.83730200	1.96404100
C	1.99701300	−1.95478200	0.44428000
O	2.92649500	−2.32846100	−0.24664600
O	−0.48758700	−2.35117900	2.82320200
H	1.48783400	−3.59362300	1.63776000
S	−0.24502200	−1.12582000	−1.75033800
O	−0.09556800	−0.85023400	−3.17250500
O	−0.21717000	−2.50296000	−1.27896800
C	−1.74244100	−0.35452600	−1.19458600
C	−2.06441100	0.91030000	−1.67918500
C	−2.56341500	−1.02322700	−0.29025100
C	−3.23218600	1.52199800	−1.22957200
H	−1.41385400	1.41139600	−2.39066500
C	−3.73059500	−0.40081200	0.13765400
H	−2.28092500	−2.00416000	0.07863000
C	−4.07902100	0.87712600	−0.32044700
H	−3.49145100	2.51248900	−1.59366600
H	−4.37830500	−0.91049900	0.84629000
C	−5.35391600	1.52834300	0.14443300
H	−5.36169400	2.59534900	−0.09014100
H	−6.21871900	1.06725800	−0.34539400
H	−5.48422100	1.40608300	1.22355000
O	2.39875600	0.37400700	0.49113200
C	2.13406700	1.69690600	0.72920800
C	3.18412900	2.42712300	1.29117700
C	0.92932700	2.32637700	0.41081200
C	3.02825900	3.78769600	1.53750500
H	4.11023300	1.91228700	1.52804300
C	0.78407400	3.68957000	0.67229600
H	0.11702100	1.76936300	−0.04130600
C	1.82515600	4.42771600	1.23217600
H	3.84966800	4.34721300	1.97580200
H	−0.15683200	4.17433300	0.42706000
H	1.70162800	5.48808800	1.42880500
Coordinates of TS3:			
C	1.36957900	−0.71061100	0.07781600
C	0.35813100	−0.89646600	1.07302000
H	−0.36992500	−0.17887900	1.41495100
N	1.50528500	−2.88419100	0.86484600
O	0.86641100	−0.51722500	−1.47570600
C	0.38413100	−2.23742500	1.50026500
C	2.13859600	−2.05675400	0.00618500
O	3.10880500	−2.28075600	−0.69138200
O	−0.32328100	−2.87377400	2.28917500
H	1.69834700	−3.87388300	0.95176700
S	−0.35303500	−1.30325700	−2.07522800
O	−0.25007000	−1.15577700	−3.52474600
O	−0.42080200	−2.65081300	−1.51785800
C	−1.78429800	−0.39041800	−1.54871000
C	−2.06876000	0.81505200	−2.18906200
C	−2.56910000	−0.85672500	−0.49908100
C	−3.15324000	1.56995300	−1.75469500
H	−1.44474100	1.15971800	−3.00854400
C	−3.65565300	−0.09052600	−0.08176100
H	−2.32437300	−1.79550300	−0.01200700
C	−3.96075200	1.12880100	−0.69750700
H	−3.37731100	2.51491700	−2.24306900
H	−4.27284100	−0.44434700	0.73992100
C	−5.15132100	1.93649100	−0.25377500
H	−4.97434500	3.00685000	−0.38840800
H	−6.03472400	1.66919600	−0.84457200
H	−5.38511900	1.74891000	0.79727600
O	2.27794300	0.32858500	0.09770600
C	1.85126100	1.60925000	0.35340500
C	2.83187500	2.48872400	0.81598300
C	0.54562000	2.05087900	0.13942200
C	2.50037900	3.81618700	1.07211400
H	3.84039800	2.11685400	0.96907100
C	0.22692300	3.38297800	0.40660700
H	−0.21237000	1.37448400	−0.23705000
C	1.19528400	4.27060400	0.87212500
H	3.26573100	4.49622100	1.43445700
H	−0.79189500	3.72270500	0.24201000
H	0.93808800	5.30503700	1.07745300
Coordinates of Product:			
C	1.90126700	−0.60859200	0.90859500
C	0.80552100	−0.80040100	1.65852000
H	0.12592400	−0.07606500	2.08533200
N	1.72086800	−2.87925200	1.17074200
O	0.82097600	−0.78761300	−1.79296500
C	0.62798600	−2.26357900	1.79640300
C	2.50294700	−1.94530500	0.52803400
O	3.47918700	−2.14465700	−0.15608300
O	−0.25571500	−2.86013100	2.37505800
H	1.72712700	−3.86392000	0.93151500
S	−0.46326900	−1.53524900	−1.80470800
O	−0.91659400	−1.88545900	−3.17020000
O	−0.47731300	−2.67093200	−0.85467200
C	−1.67879800	−0.37388500	−1.17429800
C	−2.05714900	0.71465200	−1.96315500
C	−2.19240400	−0.52389000	0.10880200
C	−2.94376500	1.65703400	−1.45375500
H	−1.65678700	0.82044200	−2.96801700
C	−3.08147800	0.43014200	0.61094700
H	−1.89651000	−1.38291400	0.70488200
C	−3.46491000	1.53248600	−0.15691200
H	−3.23825600	2.50536600	−2.06793000
H	−3.48411800	0.31124200	1.61403800
C	−4.40135700	2.57922100	0.38902100
H	−3.86543500	3.51657600	0.57740100
H	−5.20410800	2.79830300	−0.32181400
H	−4.85229600	2.25409000	1.32991700
O	2.53231100	0.48504000	0.49172300
C	1.86783400	1.69243300	0.72003100
C	2.34022100	2.53200200	1.71890800
C	0.78037500	2.02148300	−0.08164300
C	1.69225000	3.75156100	1.92411900
H	3.19556800	2.23298200	2.31681600
C	0.14198500	3.24074500	0.13909600
H	0.46551100	1.32107900	−0.85159000
C	0.59410700	4.10444000	1.13976900
H	2.04879000	4.42354600	2.69881600
H	−0.70964400	3.51543000	−0.47673900
H	0.09351200	5.05373400	1.30428800

**Table:** Below are coordinates of the reactant, TS1, and IM1 when the nucleophilic reagent is 2-hydroxy-1-naphthalenecarbaldehyde (instead of phenol):

Coordinates of Reactant:			
C	−0.75350700	−1.70159800	0.07719000
C	−0.28562100	−0.68148200	0.80264900
H	−0.57245500	0.36162700	0.82128600
N	0.90641500	−2.58477000	1.37736100
O	−1.73200100	−1.76538800	−0.86920700
C	0.81177600	−1.22613700	1.65828600
C	−0.03672700	−2.97471200	0.44237100
O	−0.28356700	−4.09784100	0.07179500
O	1.48692900	−0.62866200	2.46941600
H	1.58131600	−3.20733100	1.80540900
S	−3.27625200	−1.41538300	−0.42678000
O	−4.02113800	−1.68398500	−1.63898800
O	−3.57486900	−2.13390300	0.79840300
C	−3.20089300	0.31623200	−0.09871500
C	−2.68805400	1.15688300	−1.08790900
C	−3.63691600	0.79357400	1.13234600
C	−2.61830900	2.51726700	−0.82059900
H	−2.30318800	0.75200000	−2.01981300
C	−3.56505400	2.16454000	1.37236800
H	−4.01424000	0.11024900	1.88633200
C	−3.05712900	3.03976100	0.40664200
H	−2.20948900	3.18786600	−1.57224000
H	−3.90194800	2.55627200	2.32781400
C	−2.97877700	4.51990500	0.66381100
H	−3.24624000	4.75879600	1.69541000
H	−1.96834500	4.89293700	0.47035700
H	−3.66001500	5.05986700	−0.00244400
C	3.70828700	3.36208100	0.24368600
C	2.80188900	2.55997300	−0.42353700
C	2.91939700	1.14063500	−0.42982400
C	4.05073700	0.59526200	0.24463700
C	4.96644700	1.43406200	0.91417700
C	4.80348900	2.80500900	0.93033000
H	3.57685000	4.44073600	0.22205500
H	2.00032900	3.04567200	−0.96795900
C	1.98907200	0.25968200	−1.10517400
C	4.26053500	−0.82660200	0.21022100
H	5.81402100	0.97302500	1.41717700
H	5.51283000	3.44346700	1.44792600
C	3.40818500	−1.65209600	−0.44226400
C	2.21143000	−1.16528900	−1.13501400
H	5.13333000	−1.22704500	0.72398400
H	3.56760200	−2.72786600	−0.46459700
C	0.77585900	0.81521600	−1.67351400
H	0.50856900	1.82640000	−1.30353000
O	0.01378800	0.29100200	−2.48328000
O	1.42426400	−2.00245500	−1.64592700
Coordinates of TS1:			
C	−0.41337900	−1.23791500	−0.05310100
C	−0.30728100	−0.10829500	0.73988800
H	−0.68729100	0.88400800	0.55050700
N	0.77413700	−1.84406800	1.80590100
O	−1.44425100	−1.48878500	−0.96031800
C	0.49675900	−0.45108100	1.88583100
C	0.11904300	−2.42140600	0.74986300
O	0.01029100	−3.60234600	0.49972300
O	0.90072900	0.23638200	2.81572800
H	1.22262100	−2.36381500	2.55094900
S	−2.95906600	−1.58194900	−0.37661600
O	−3.70538600	−2.11271800	−1.50231500
O	−2.94413500	−2.28851800	0.89246100
C	−3.40981600	0.10524900	−0.10401500
C	−3.44458900	0.97289800	−1.19599400
C	−3.71071600	0.53015500	1.18440000
C	−3.78663700	2.30051300	−0.97685800
H	−3.19766800	0.62015300	−2.19328300
C	−4.06048600	1.86496400	1.38002000
H	−3.66240500	−0.16523500	2.01633300
C	−4.09957200	2.76433400	0.31029800
H	−3.81058700	2.99109500	−1.81580800
H	−4.29859400	2.21151000	2.38169500
C	−4.46847300	4.20785000	0.52091100
H	−4.68442200	4.41249300	1.57182400
H	−3.65228600	4.86388500	0.20161100
H	−5.35080300	4.47114800	−0.07123500
C	5.02871500	2.77191100	0.18494900
C	3.80137800	2.34784300	−0.27699000
C	3.50672500	0.96245900	−0.42408300
C	4.54193400	0.03359000	−0.11431300
C	5.79710700	0.49750600	0.35032800
C	6.04169300	1.84285200	0.50799600
H	5.22030500	3.83586800	0.29218300
H	3.05976500	3.09460800	−0.53906400
C	2.23936800	0.47249300	−0.90374100
C	4.29905200	−1.36317600	−0.29291300
H	6.56720800	−0.23626100	0.57746600
H	7.00539600	2.19069800	0.86719300
C	3.08983300	−1.81493000	−0.72841300
C	2.01954900	−0.90697500	−1.04467000
H	5.09983200	−2.06218500	−0.06140000
H	2.88840000	−2.87678800	−0.84411800
C	1.17231400	1.43489400	−1.24171600
H	1.13244900	2.34382300	−0.61153900
O	0.37267300	1.31407700	−2.15504700
O	0.85496100	−1.41181200	−1.36958500
Coordinates of IM1:			
C	−0.19913900	−1.08098600	−0.14826300
C	−0.33278100	0.02738000	0.78336000
H	−0.67032800	1.02234300	0.53509600
N	0.22800100	−1.83300900	2.01550500
O	−1.35796300	−1.41604600	−0.99096500
C	−0.12513700	−0.42622800	2.08801100
C	0.14165900	−2.31038500	0.75800400
O	0.31771200	−3.44704600	0.35593600
O	−0.18666400	0.12209700	3.19938300
H	0.36059000	−2.40923600	2.83751400
S	−2.81879400	−1.53808600	−0.34415400
O	−3.61035200	−2.12645200	−1.41392000
O	−2.73712700	−2.21360300	0.94221500
C	−3.35839600	0.13111500	−0.09705300
C	−3.49728600	0.95950500	−1.20958100
C	−3.63854400	0.57602500	1.18888000
C	−3.91915400	2.26905200	−1.01509400
H	−3.26802600	0.59229800	−2.20592900
C	−4.07024400	1.88954000	1.36132500
H	−3.50320200	−0.08727100	2.03747400
C	−4.21250800	2.75112300	0.26908000
H	−4.02222800	2.93051800	−1.87155700
H	−4.28869300	2.25132300	2.36226800
C	−4.67605700	4.17119600	0.45492300
H	−4.80088700	4.41061700	1.51342800
H	−3.95629000	4.87386700	0.02362500
H	−5.63473300	4.33319800	−0.04895400
C	5.49943800	2.67832700	−0.06674700
C	4.19580100	2.38969900	−0.39347900
C	3.74311300	1.04107900	−0.46053400
C	4.68118500	−0.00096800	−0.20607200
C	6.02333600	0.32972900	0.12343300
C	6.42606600	1.64043400	0.19758000
H	5.82484900	3.71328600	−0.01868100
H	3.51237900	3.20380900	−0.61336600
C	2.39607000	0.69724600	−0.80564100
C	4.25687200	−1.35452100	−0.29730400
H	6.72500700	−0.47890700	0.31209300
H	7.45320500	1.88530400	0.45030100
C	2.96365900	−1.66605700	−0.62679900
C	2.02968500	−0.63270900	−0.88627200
H	4.97896900	−2.14332200	−0.10283800
H	2.62194300	−2.69419300	−0.69479300
C	1.38870600	1.77486000	−1.01439600
H	1.40810200	2.59357800	−0.27175100
O	0.59152300	1.80114800	−1.92977200
O	0.75450900	−0.99419200	−1.22564800

## Appendix A Supplementary data

The ^1^H and ^13^C NMR spectra of the aryloxymaleimide compounds and the reactant tosyloxymaleimide are available in the Supplementary Information.
