# Supplementary material for: DFT calculation and NMR data of novel aryloxymaleimides and the intermediates and transition states in the reaction
Source: Data Brief. 2019 Jun 8;25:104110. doi: 10.1016/j.dib.2019.104110 (PMC6597784; doi:10.1016/j.dib.2019.104110)
Supplement: Supplementary file 1 [file mmc1.pdf]

^{1}H and ^{13}C NMR spectra of tosyloxymaleimide and aryloxymaleimide compounds

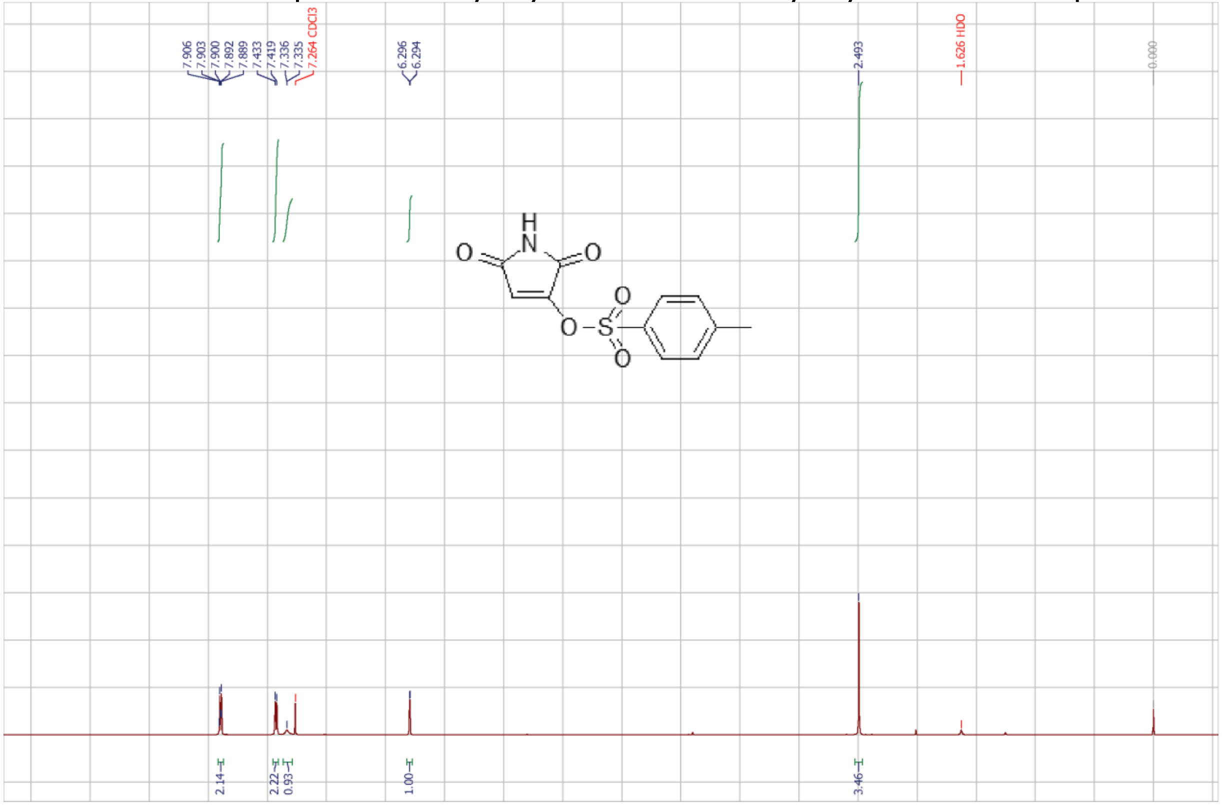

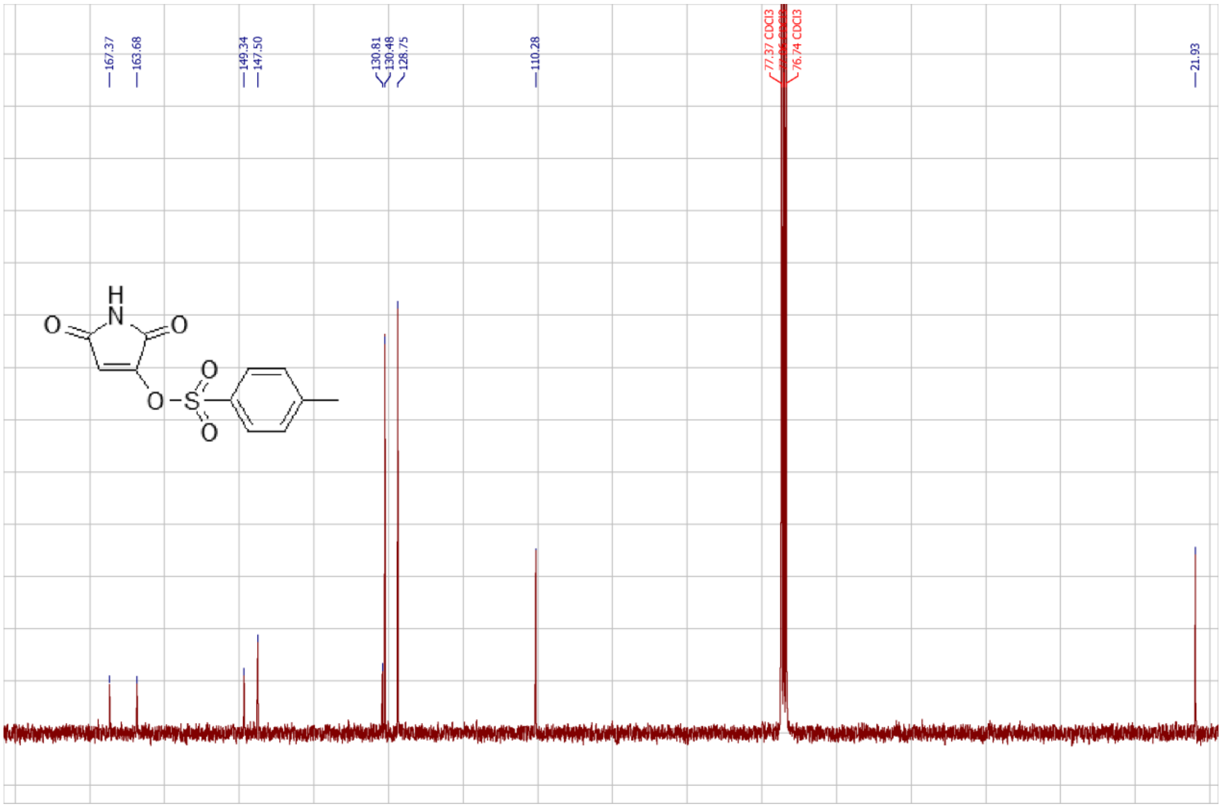

PH.10.1.1r
1H NMR PH in CDCl3

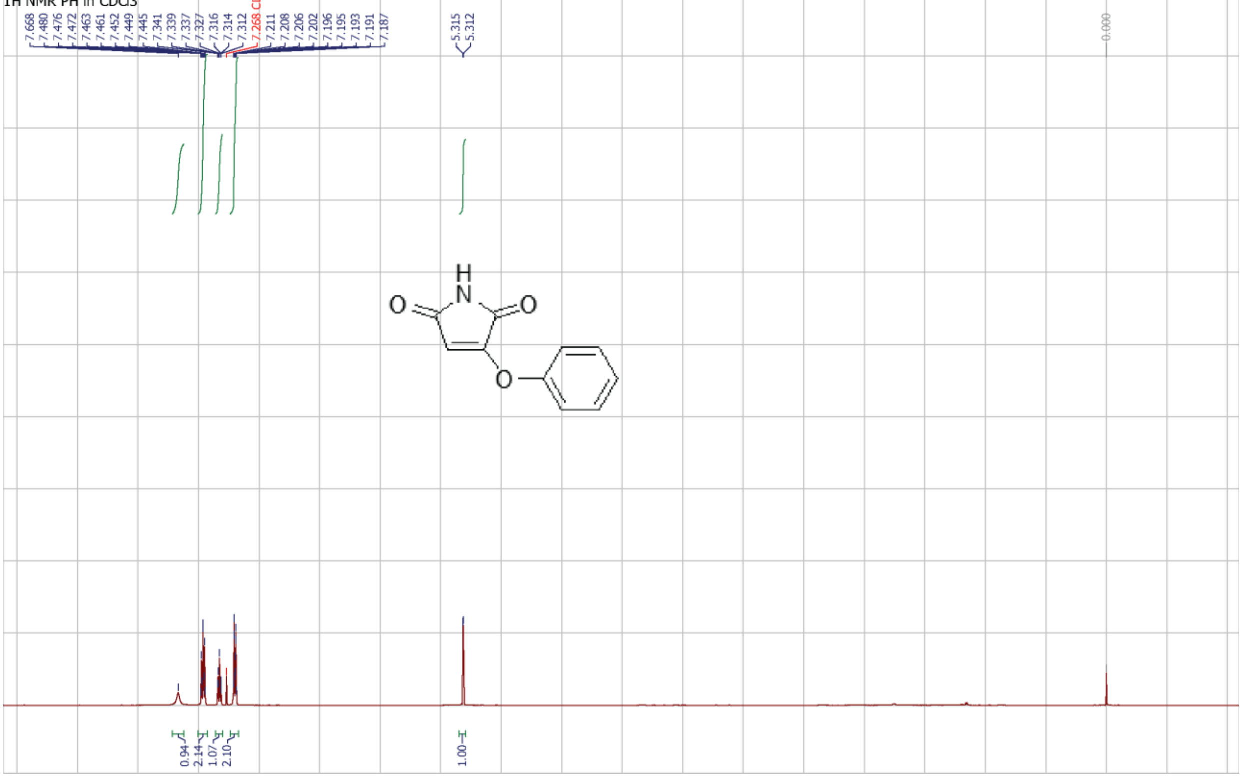

lzh0124.20.1.1r

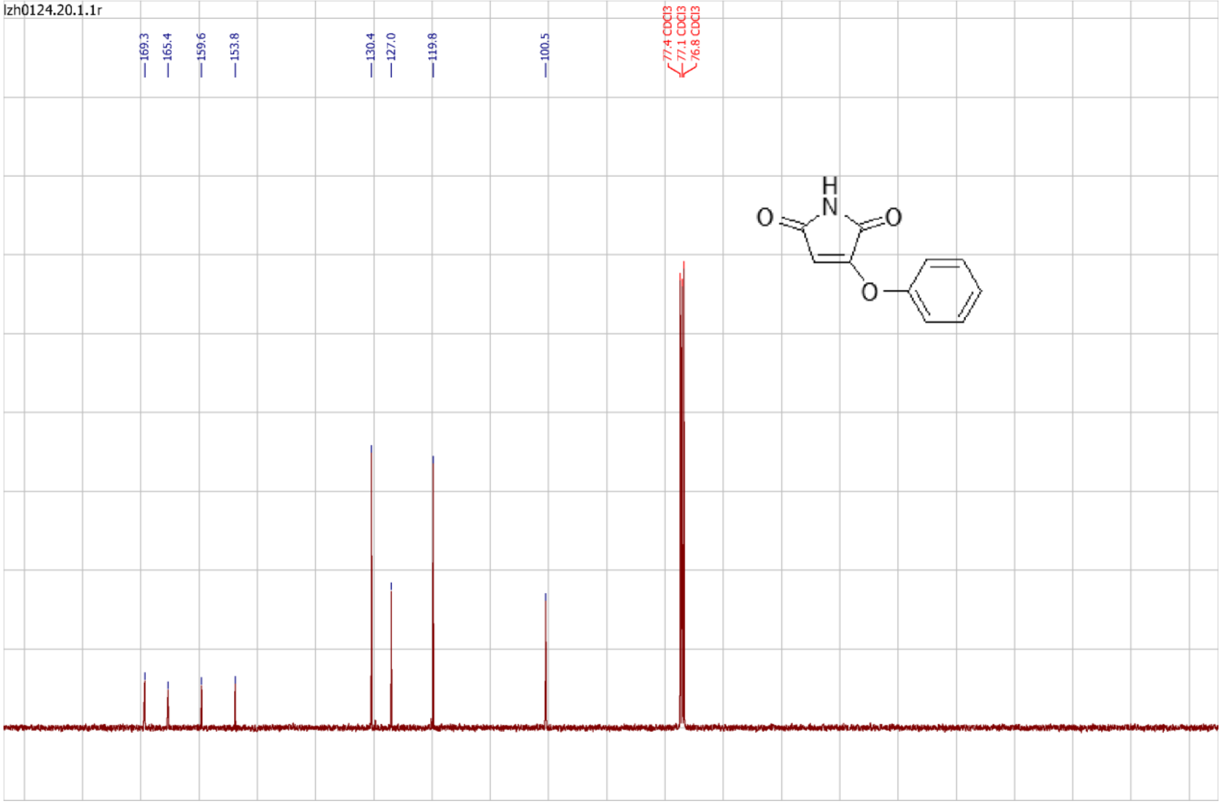

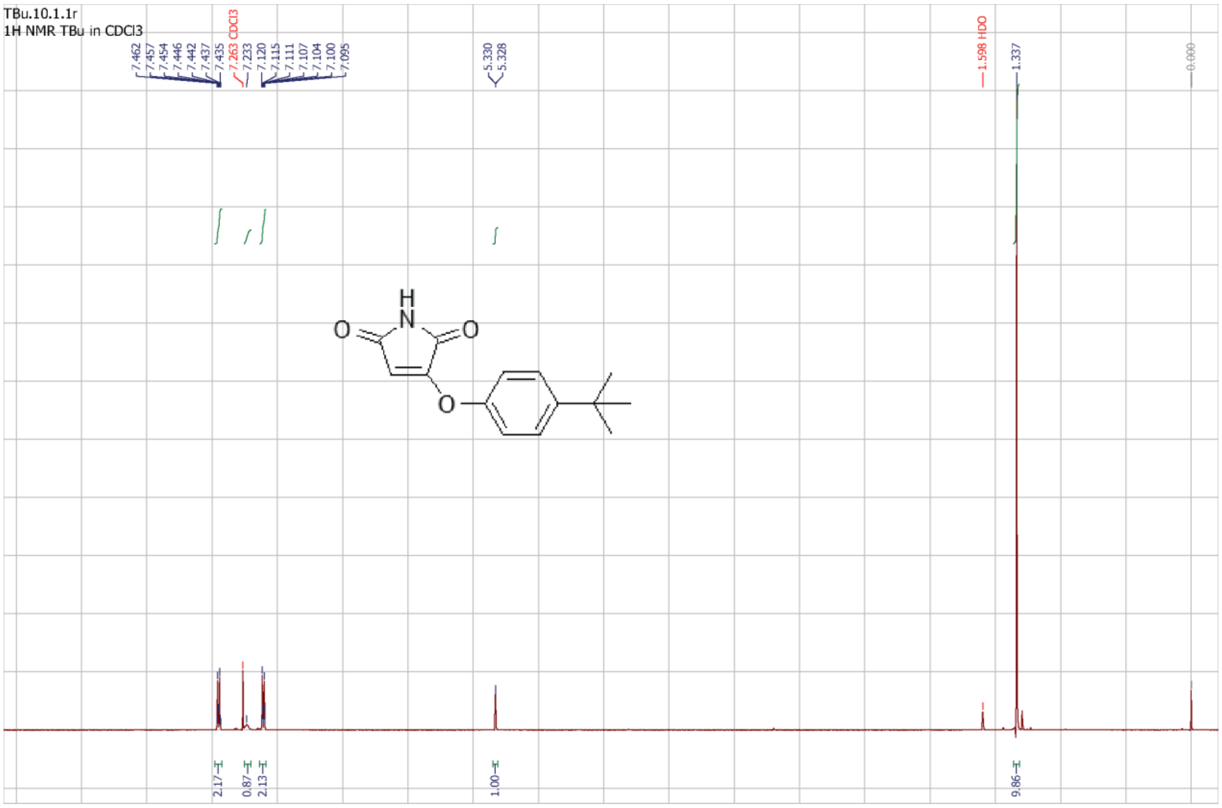
TBu.10.1.1r
1H NMR TBu in CDCl3
7.462
7.457
7.454
7.446
7.442
7.437
7.435
7.263 CDCl3
7.233
7.120
7.115
7.111
7.107
7.104
7.100
7.095
5.330
5.328
1.598 HDO
1.337
0.000
2.17
0.87
2.13
1.00
9.86
H
N
O
O
O

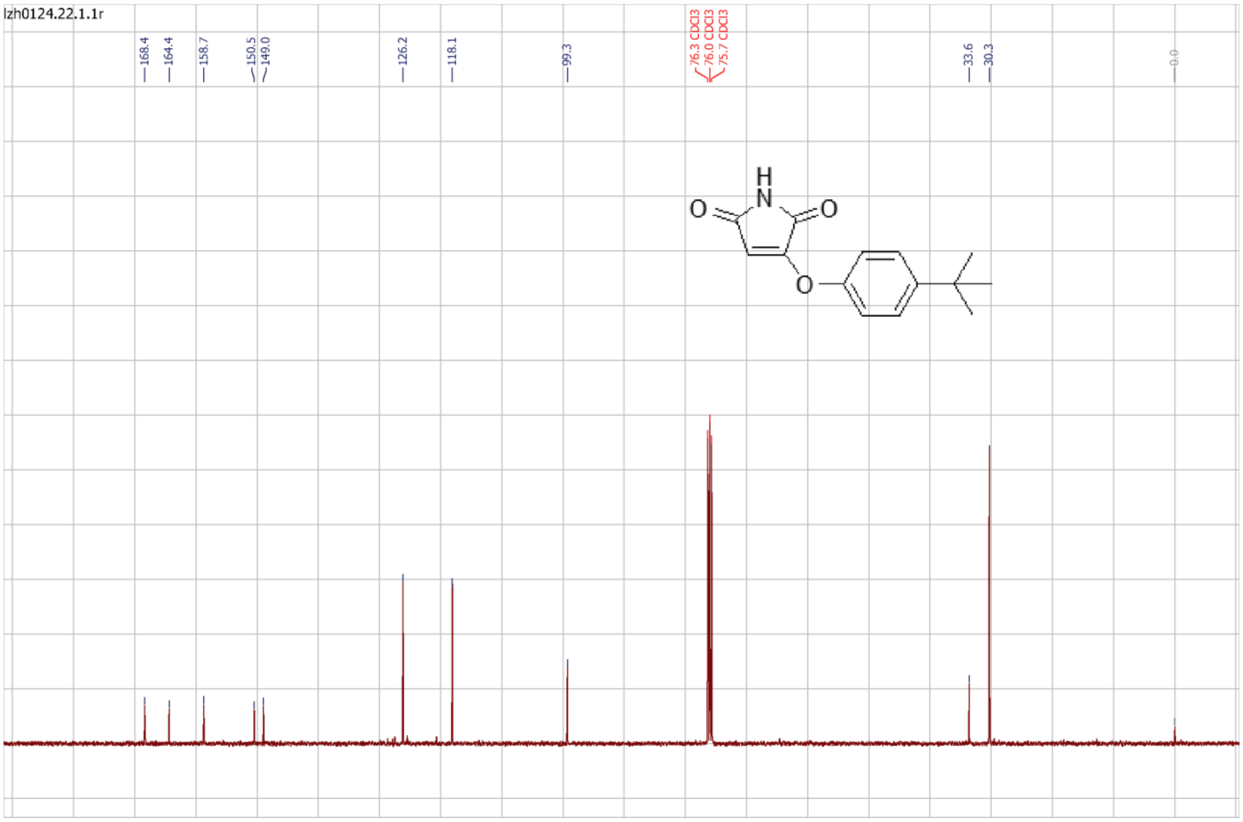
lzh0124.22.1.1r
168.4
164.4
158.7
150.5
149.0
126.2
118.1
99.3
76.3 CDCl3
76.0 CDCl3
75.7 CDCl3
33.6
30.3
0.0
H
N
O
O
O

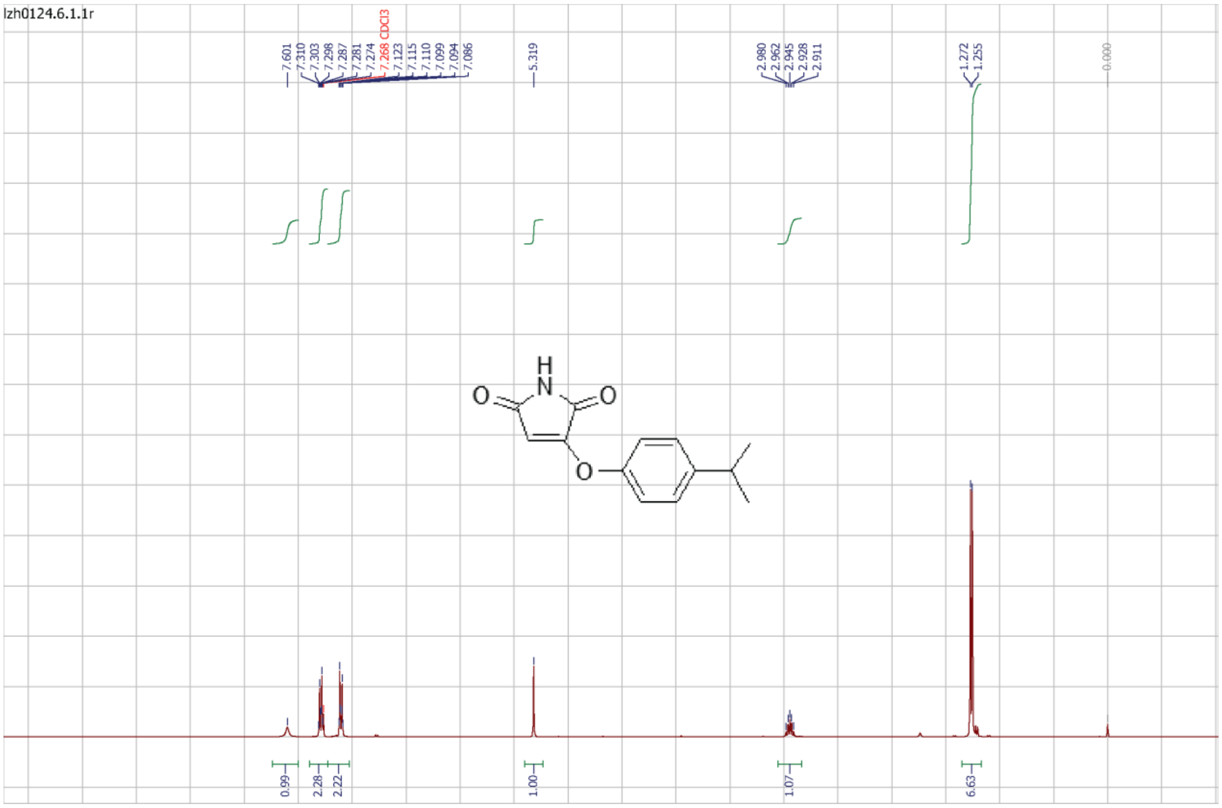
lzh0124.6.1.1r
7.601
7.310
7.303
7.298
7.287
7.281
7.274
7.268 CDCl3
7.123
7.115
7.110
7.099
7.094
7.086
5.319
2.980
2.962
2.945
2.928
2.911
1.272
1.255
0.000
H
N
O
O
O
0.99
2.28
2.22
1.00
1.07
6.63

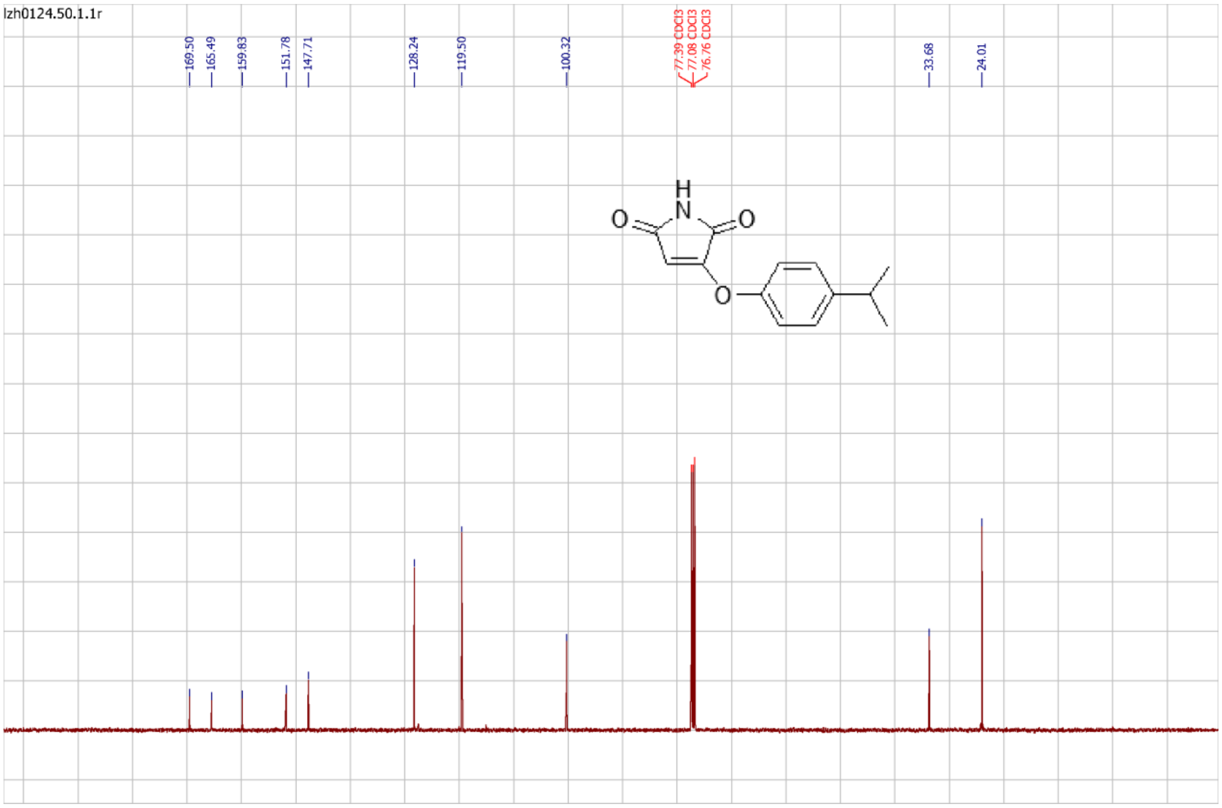
lzh0124.50.1.1r
169.50
165.49
159.83
151.78
147.71
128.24
119.50
100.32
77.39 CDCl3
77.08 CDCl3
76.76 CDCl3
33.68
24.01
H
N
O
O
O

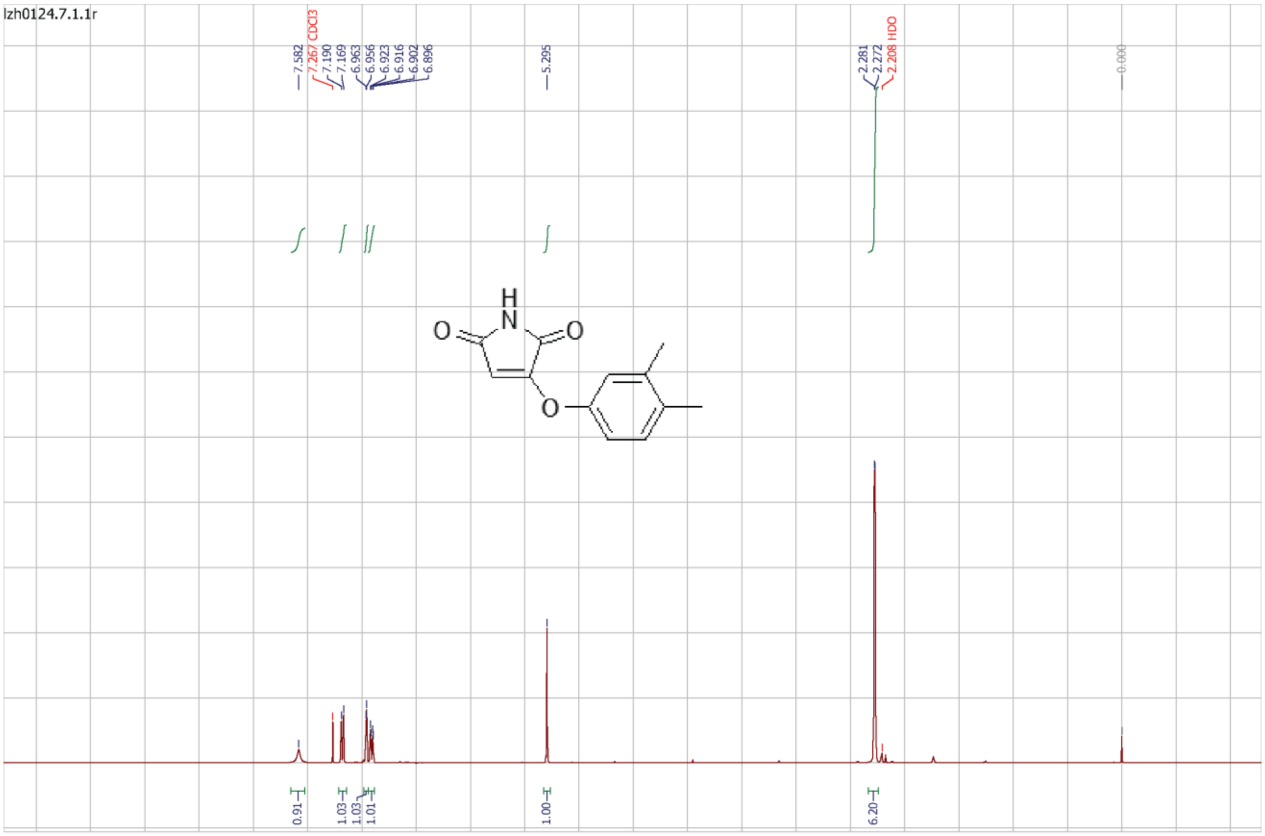
lzh0124.7.1.1r
7.582
7.267 CDCl3
7.190
7.169
6.963
6.956
6.923
6.916
6.902
6.896
5.295
2.281
2.272
2.208 HDO
0.000
0.91
1.03
1.03
1.01
1.00
6.20

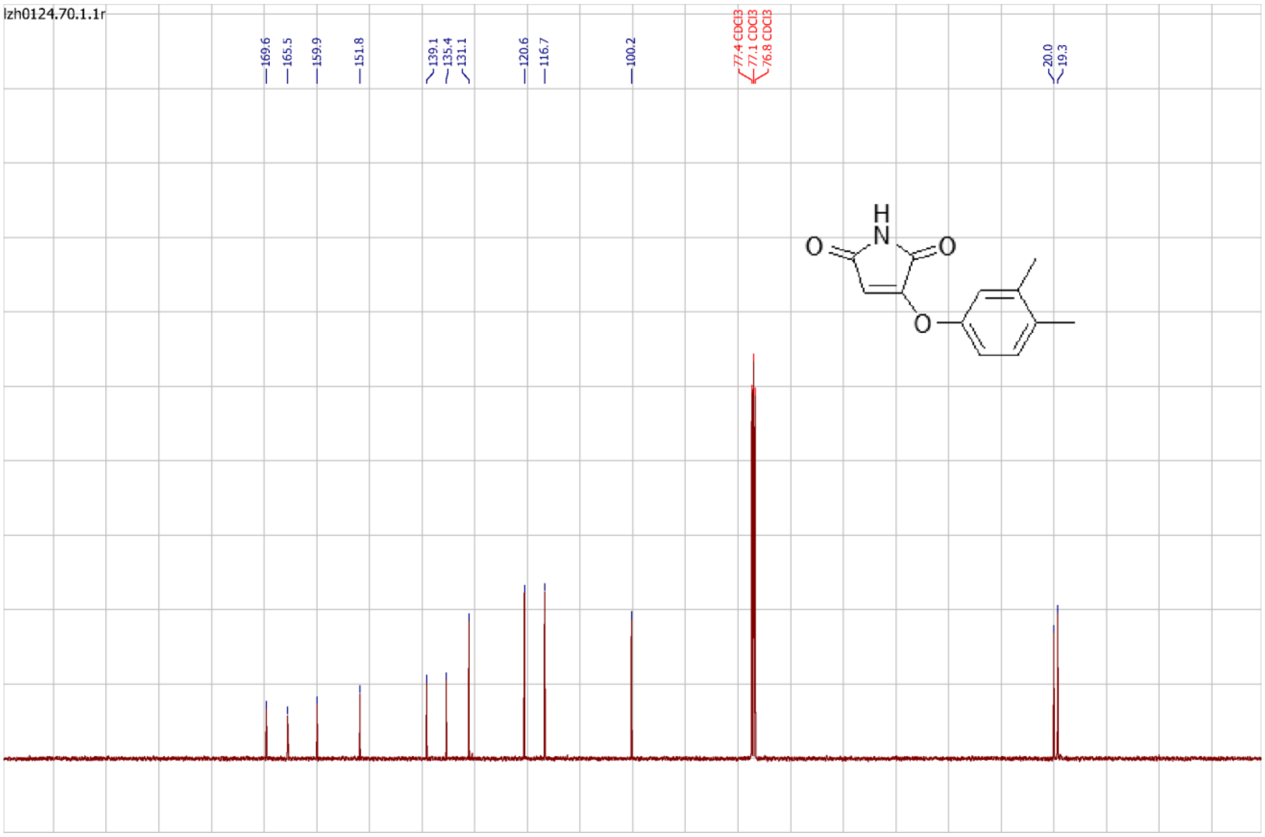
lzh0124.70.1.1r
169.6
165.5
159.9
151.8
139.1
135.4
131.1
120.6
116.7
100.2
77.4 CDCl3
77.1 CDCl3
76.8 CDCl3
20.0
19.3

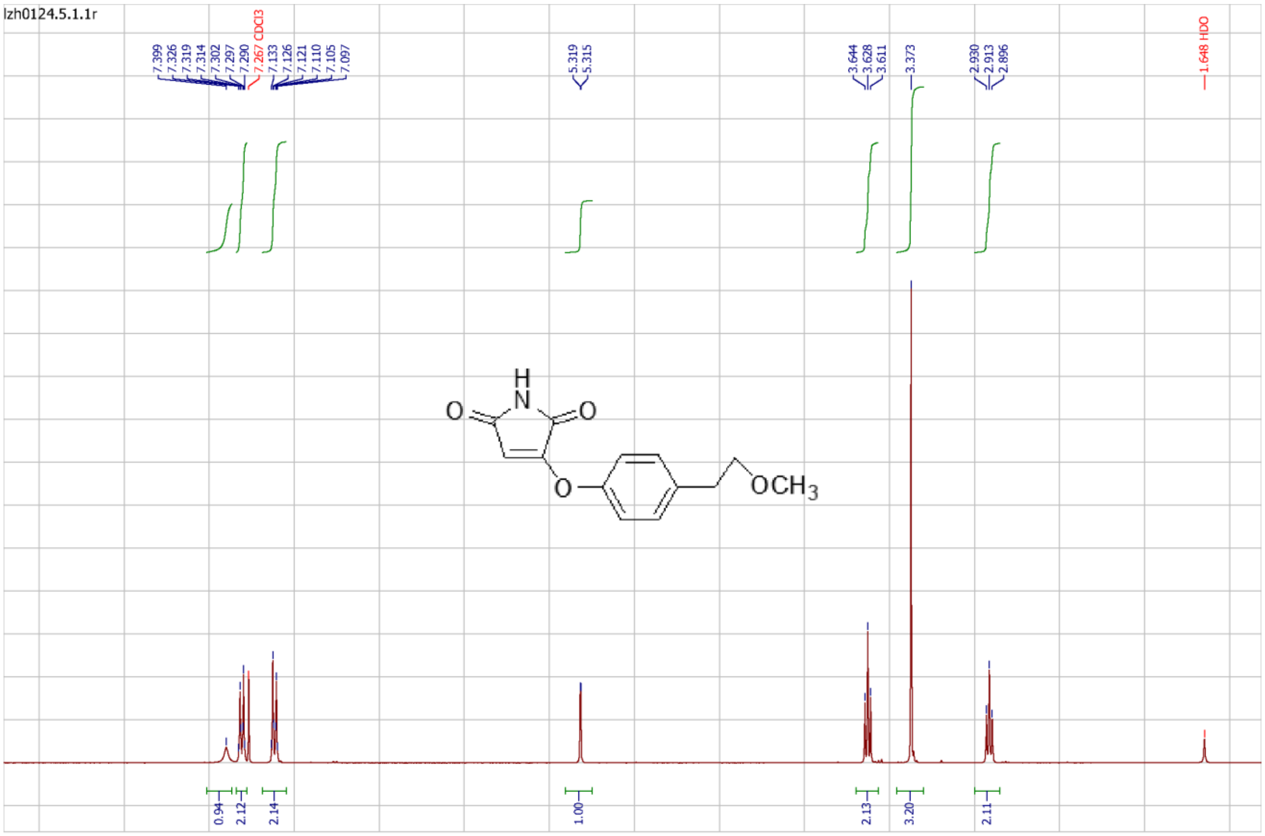
lzh0124.5.1.1r
7.399
7.326
7.319
7.314
7.302
7.297
7.290
7.267 CDCl3
7.133
7.126
7.121
7.110
7.105
7.097
5.319
5.315
3.644
3.628
3.611
3.373
2.930
2.913
2.896
1.648 HDO
0.94
2.12
2.14
1.00
2.13
3.20
2.11
H
N
O
O
O
OCH3

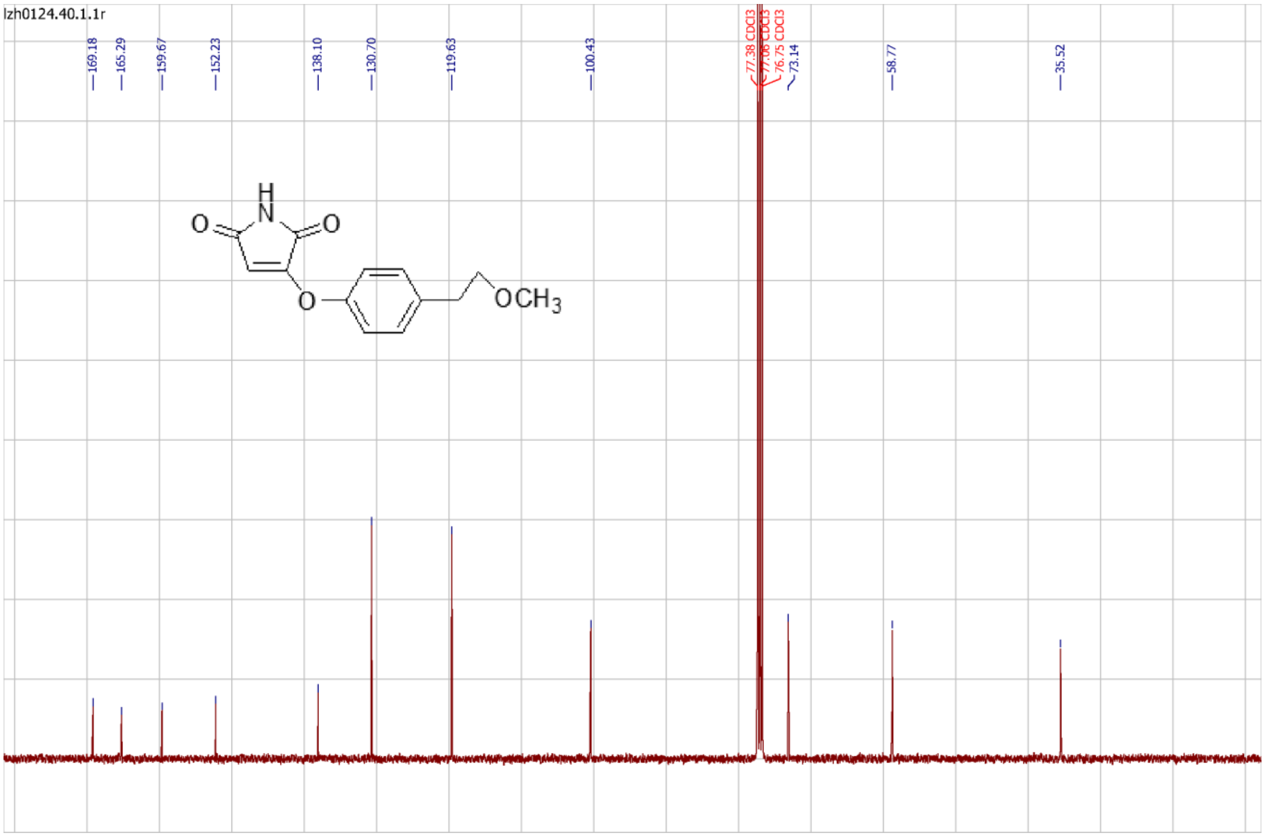
lzh0124.40.1.1r
169.18
165.29
159.67
152.23
138.10
130.70
119.63
100.43
77.38 CDCl3
77.06 CDCl3
76.75 CDCl3
73.14
58.77
35.52
H
N
O
O
O
OCH3

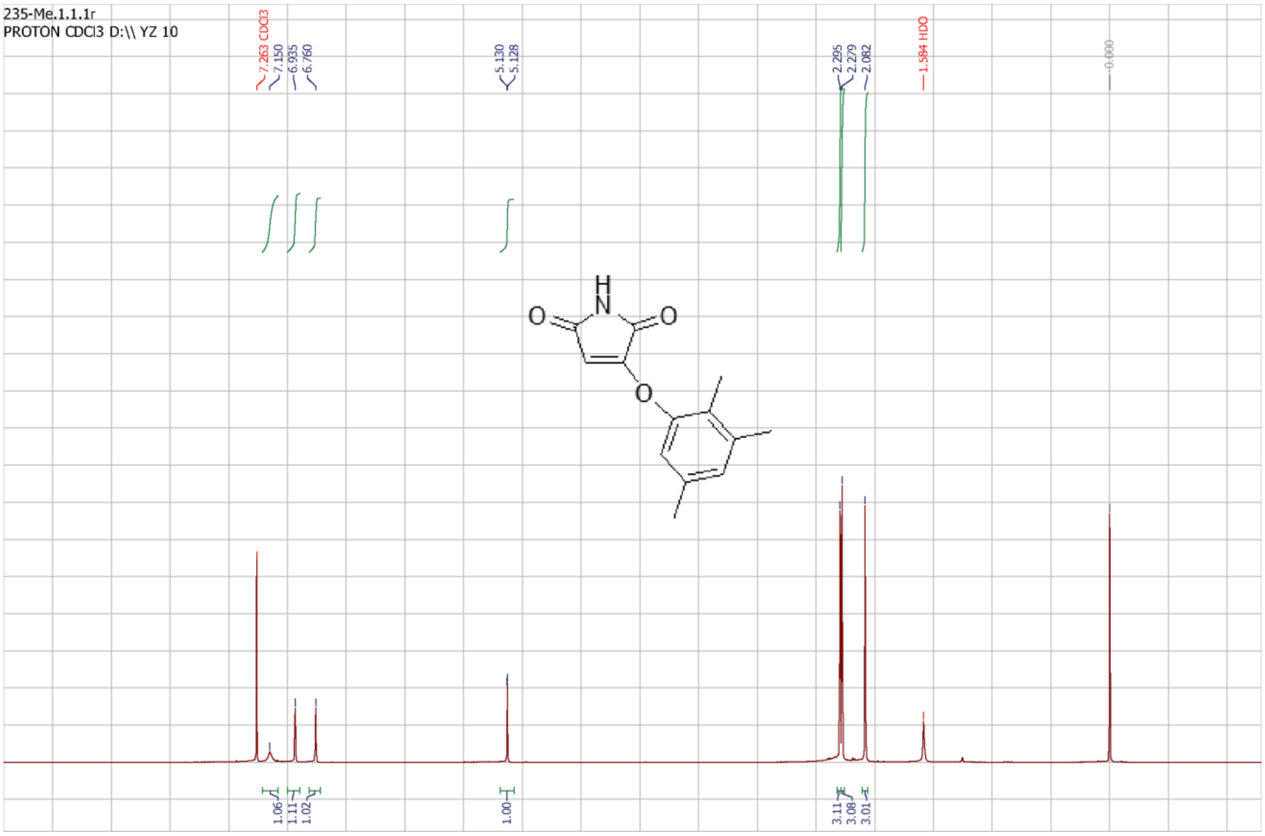
235-Me.1.1.1r
PROTON CDCl3 D:\\ YZ 10
7.263 CDCl3
7.150
6.935
6.760
5.130
5.128
2.295
2.279
2.082
1.584 HDO
0.000
1.06
1.11
1.02
1.00
3.11
3.08
3.01

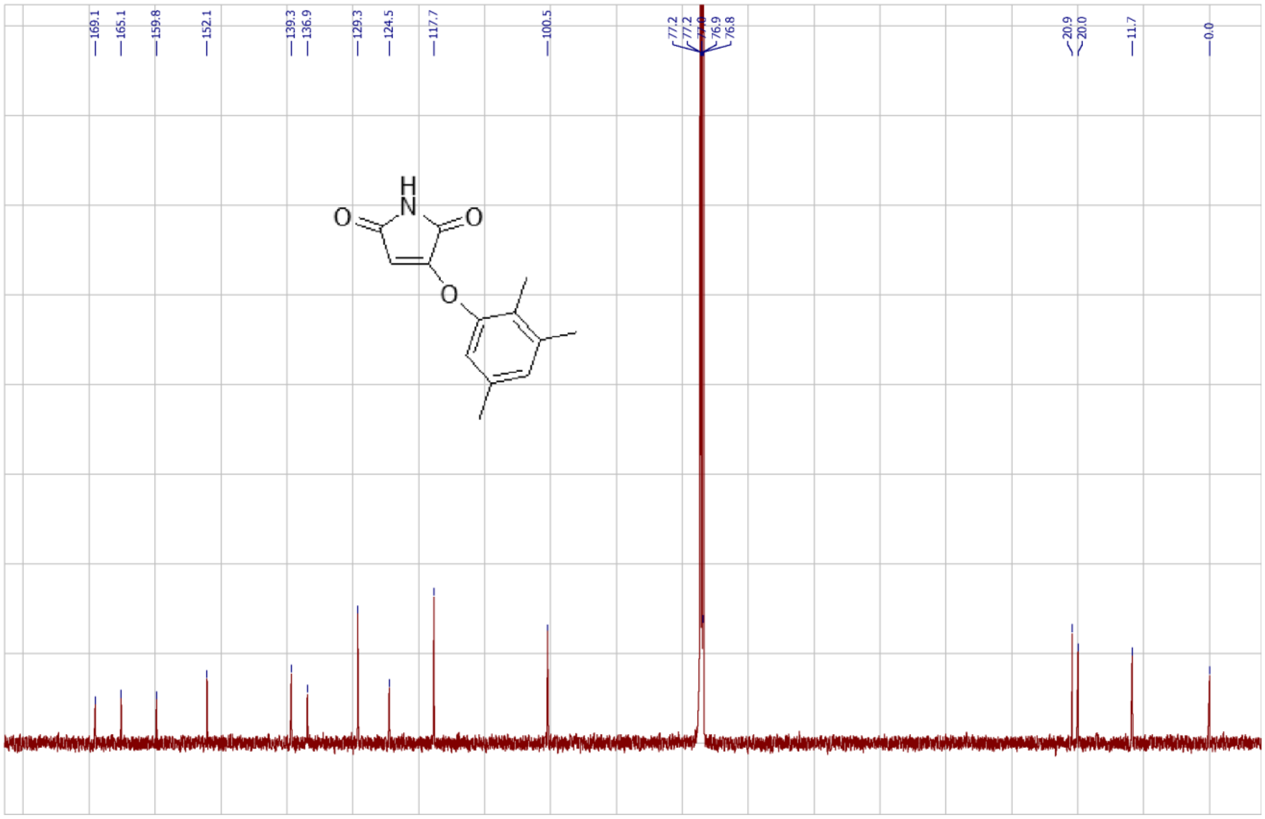
169.1
165.1
159.8
152.1
139.3
136.9
129.3
124.5
117.7
100.5
77.2
77.2
76.9
76.8
20.9
20.0
11.7
0.0

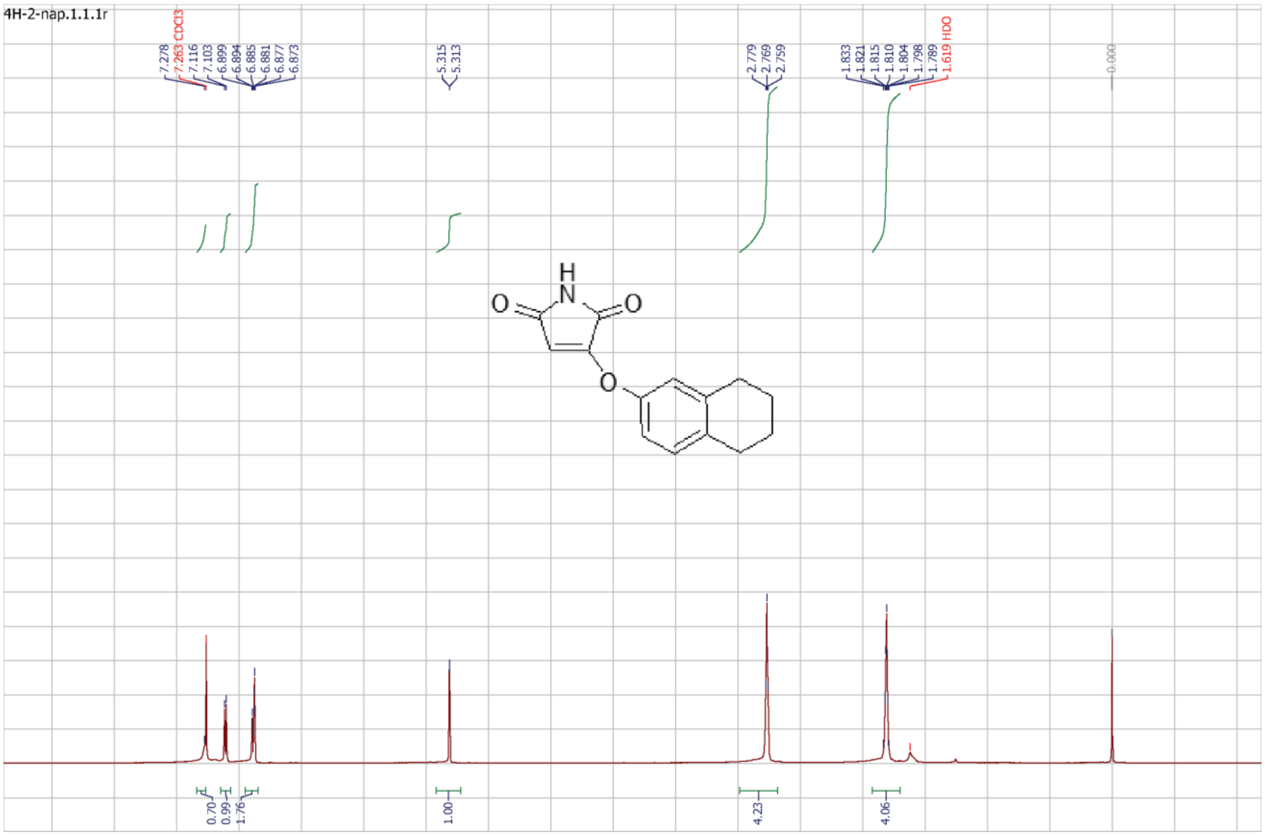
4H-2-nap.1.1.1r
7.278
7.263 CDCl3
7.116
7.103
6.899
6.894
6.885
6.881
6.877
6.873
5.315
5.313
2.779
2.769
2.759
1.833
1.821
1.815
1.810
1.804
1.798
1.789
1.619 HDO
0.000
0.70
0.99
1.76
1.00
4.23
4.06
H
N
O
O
O

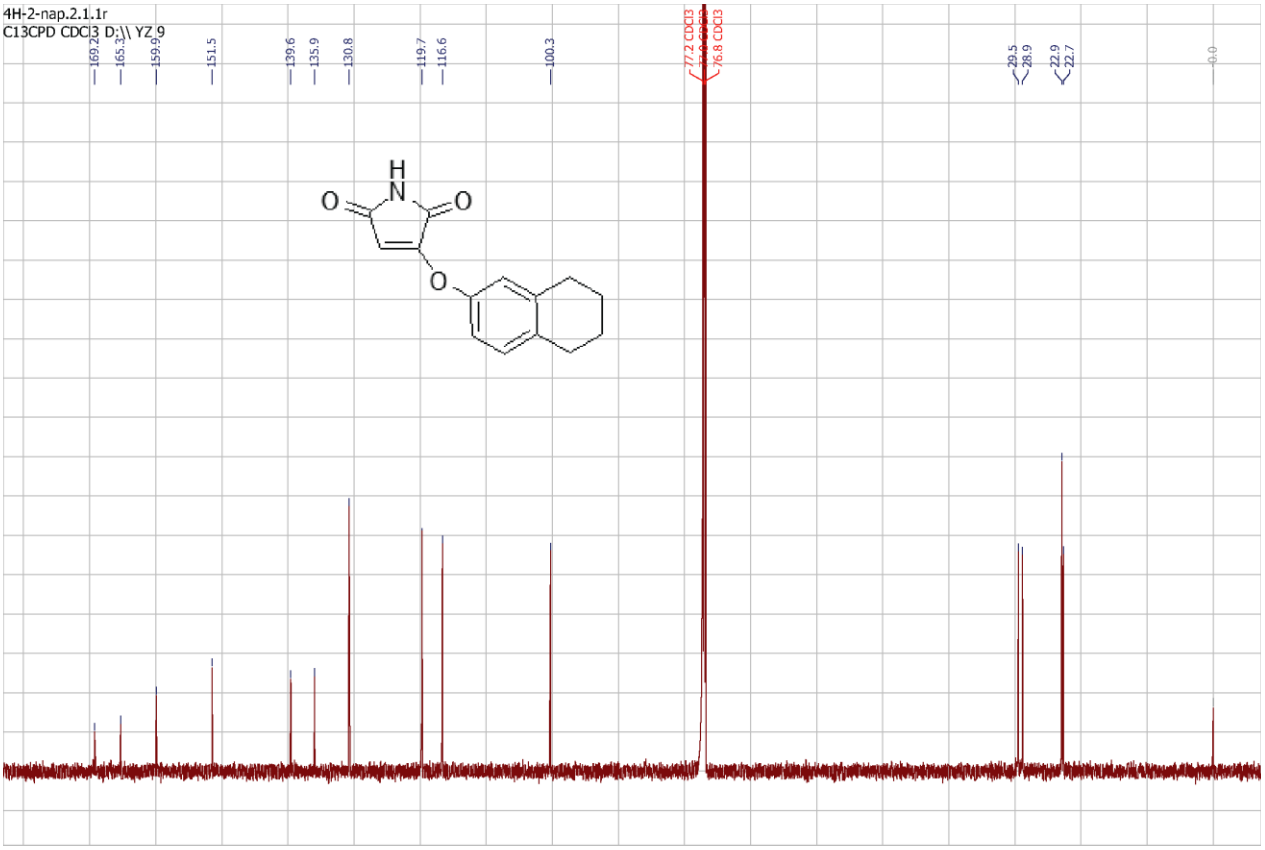
4H-2-nap.2.1.1r
C13CPD CDCl3 D:\\ YZ 9
169.2
165.3
159.9
151.5
139.6
135.9
130.8
119.7
116.6
100.3
77.2 CDCl3
77.0 CDCl3
76.8 CDCl3
29.5
28.9
22.9
22.7
0.0
H
N
O
O
O

1-nap.1.1.1r
PROTON CDCl3 D:\\ YZ 11

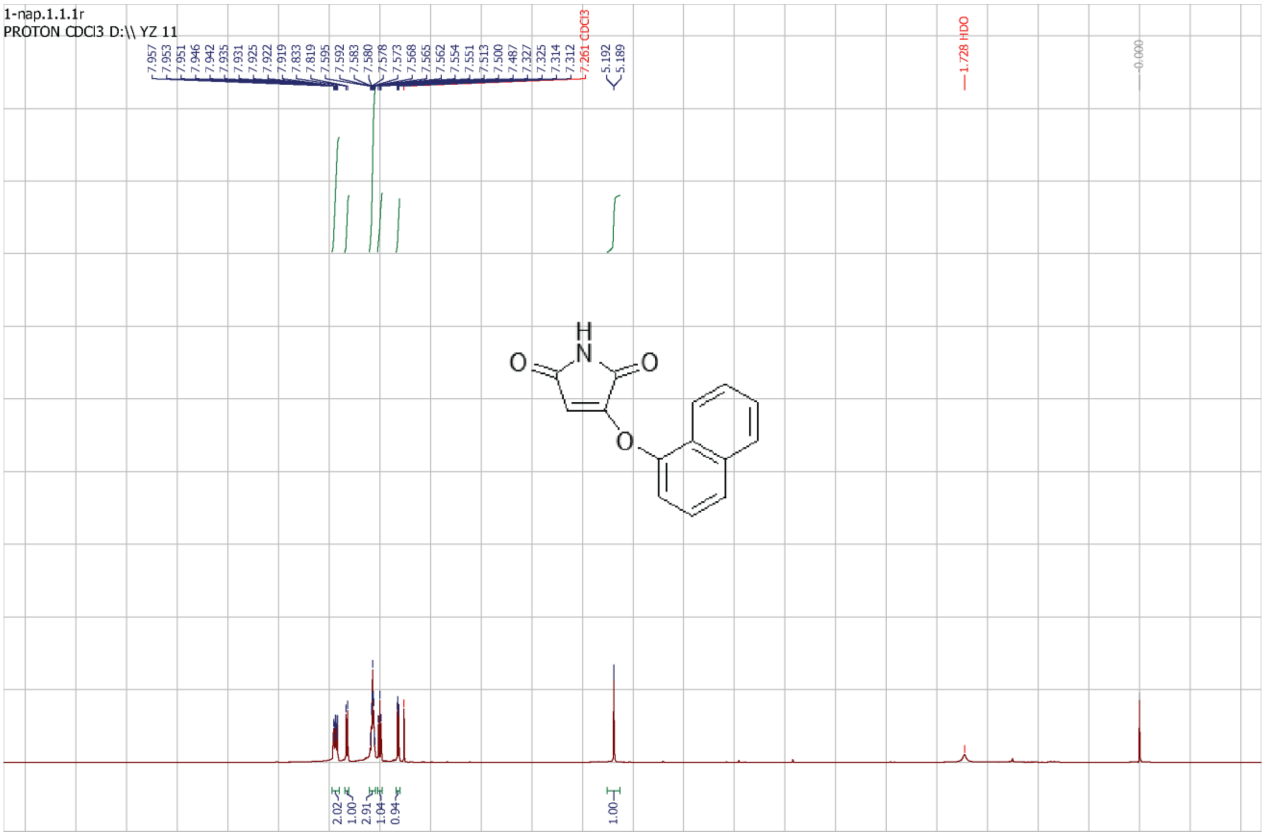

1-nap.2.1.1r
C13CPD CDCl3 D:\\ YZ 11

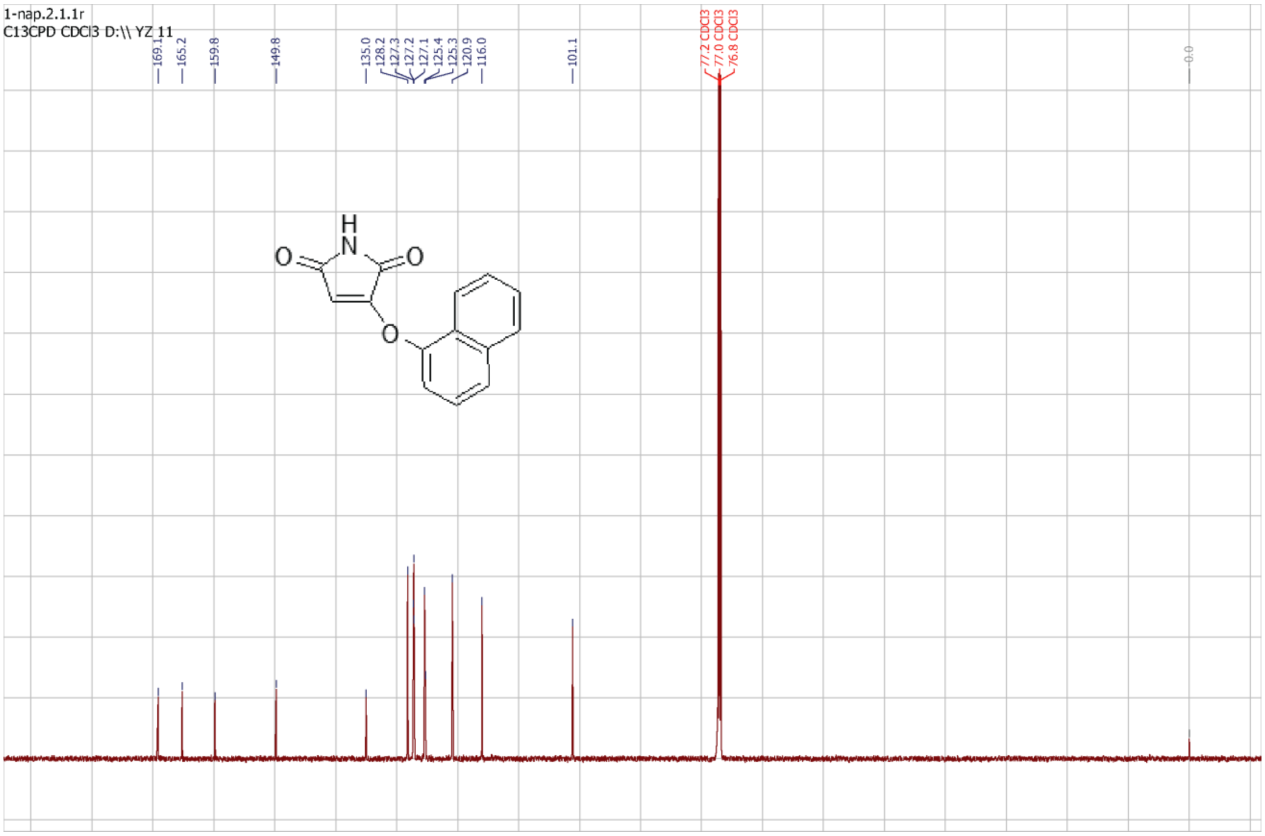

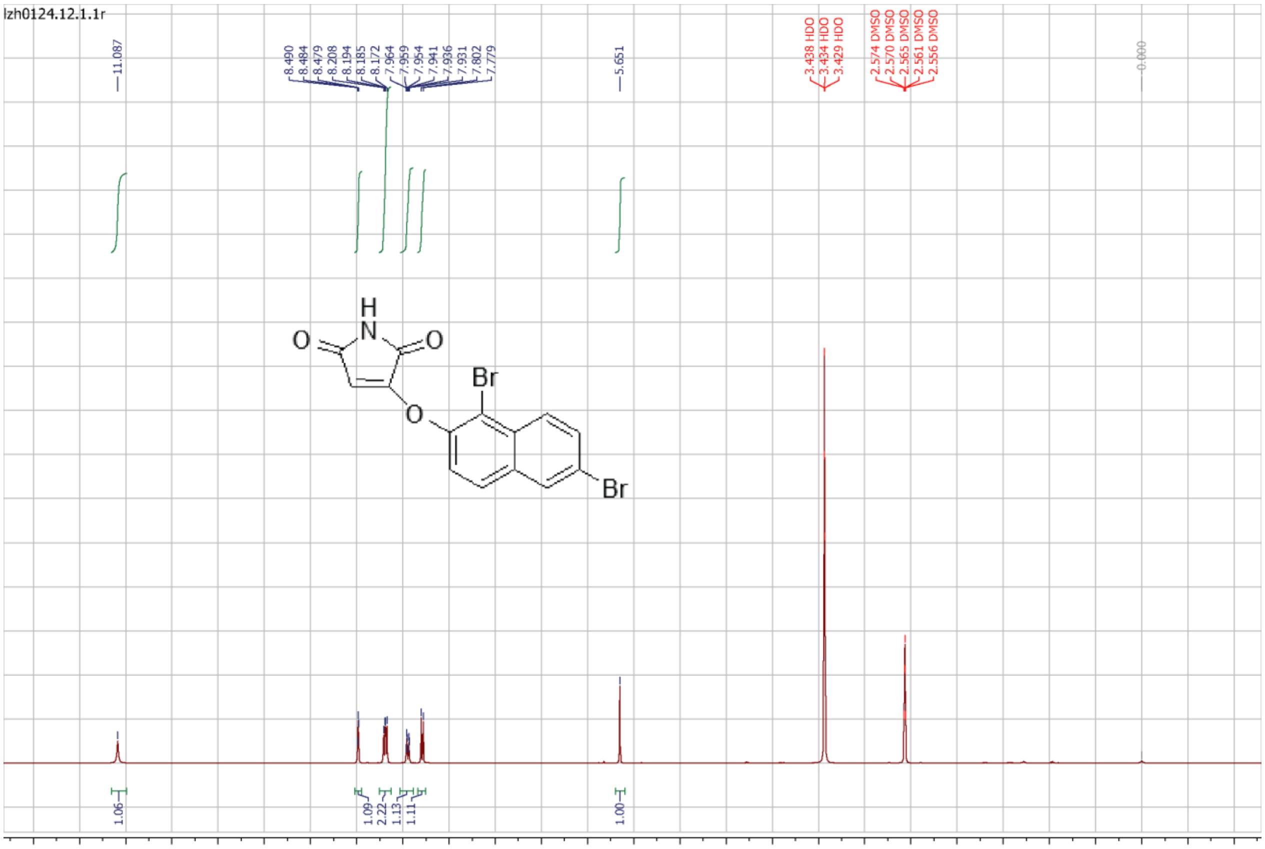
lzh0124.12.1.1r
11.087
8.490
8.484
8.479
8.208
8.194
8.185
8.172
7.964
7.959
7.954
7.941
7.936
7.931
7.802
7.779
5.651
3.438 HDO
3.434 HDO
3.429 HDO
2.574 DMSO
2.570 DMSO
2.565 DMSO
2.561 DMSO
2.556 DMSO
0.000
1.06
1.09
2.22
1.13
1.11
1.00
H
N
O
O
Br
O
Br

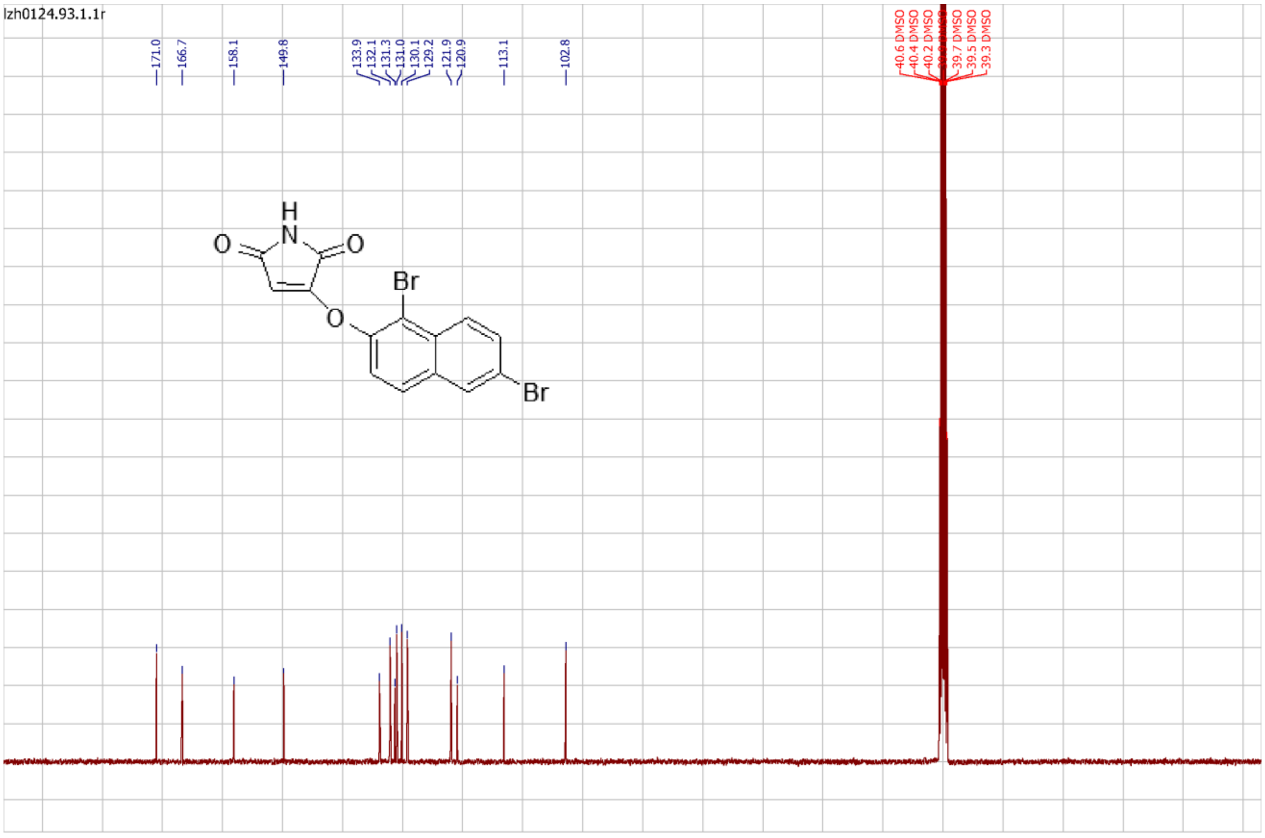
lzh0124.93.1.1r
171.0
166.7
158.1
149.8
133.9
132.1
131.3
131.0
130.1
129.2
121.9
120.9
113.1
102.8
40.6 DMSO
40.4 DMSO
40.2 DMSO
39.7 DMSO
39.5 DMSO
39.3 DMSO
H
N
O
O
Br
O
Br

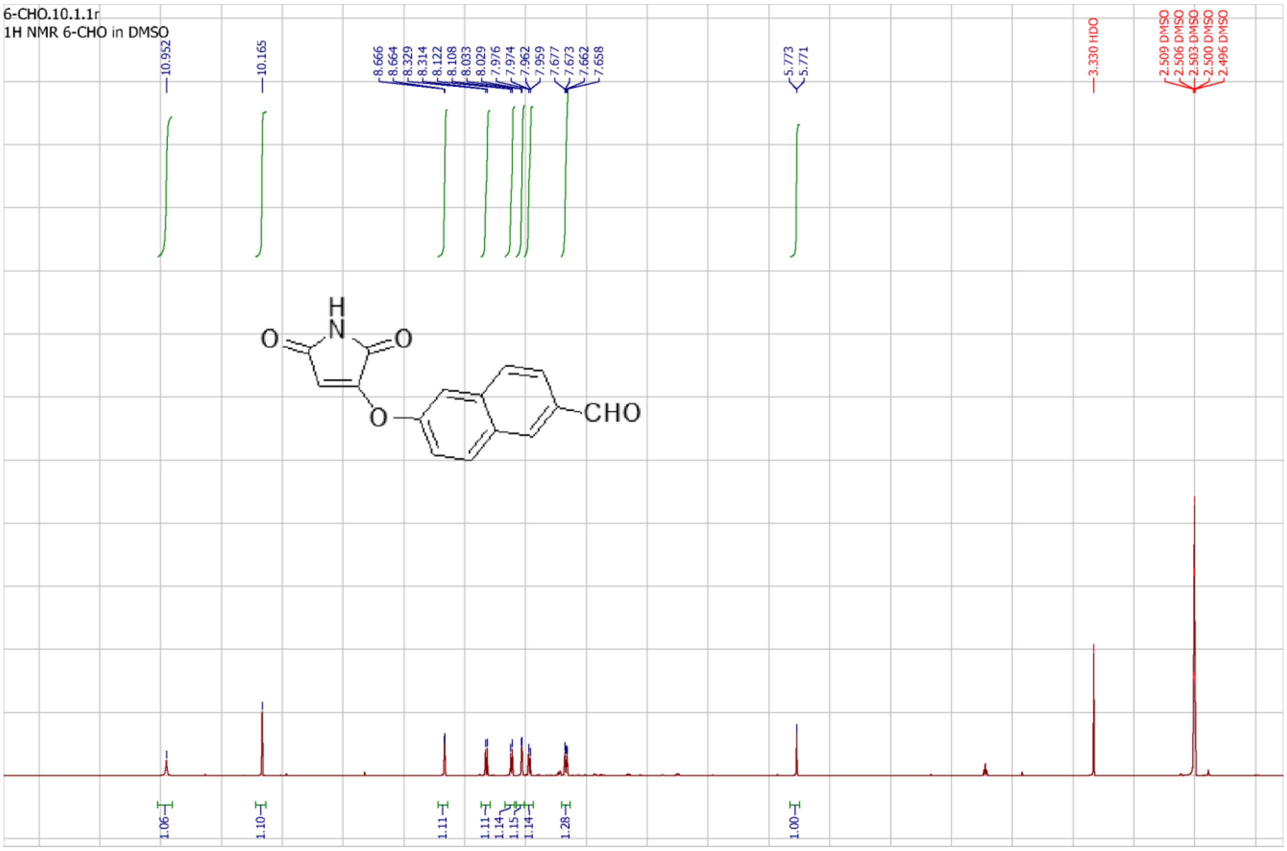

6-CHO.10.1.1r
1H NMR 6-CHO in DMSO
10.952
10.165
8.666
8.664
8.329
8.314
8.122
8.108
8.033
8.029
7.976
7.974
7.962
7.959
7.677
7.673
7.662
7.658
5.773
5.771
3.330 HDO
2.509 DMSO
2.506 DMSO
2.503 DMSO
2.500 DMSO
2.496 DMSO
H
N
O
O
O
CHO
1.06
1.10
1.11
1.11
1.14
1.15
1.14
1.28
1.00

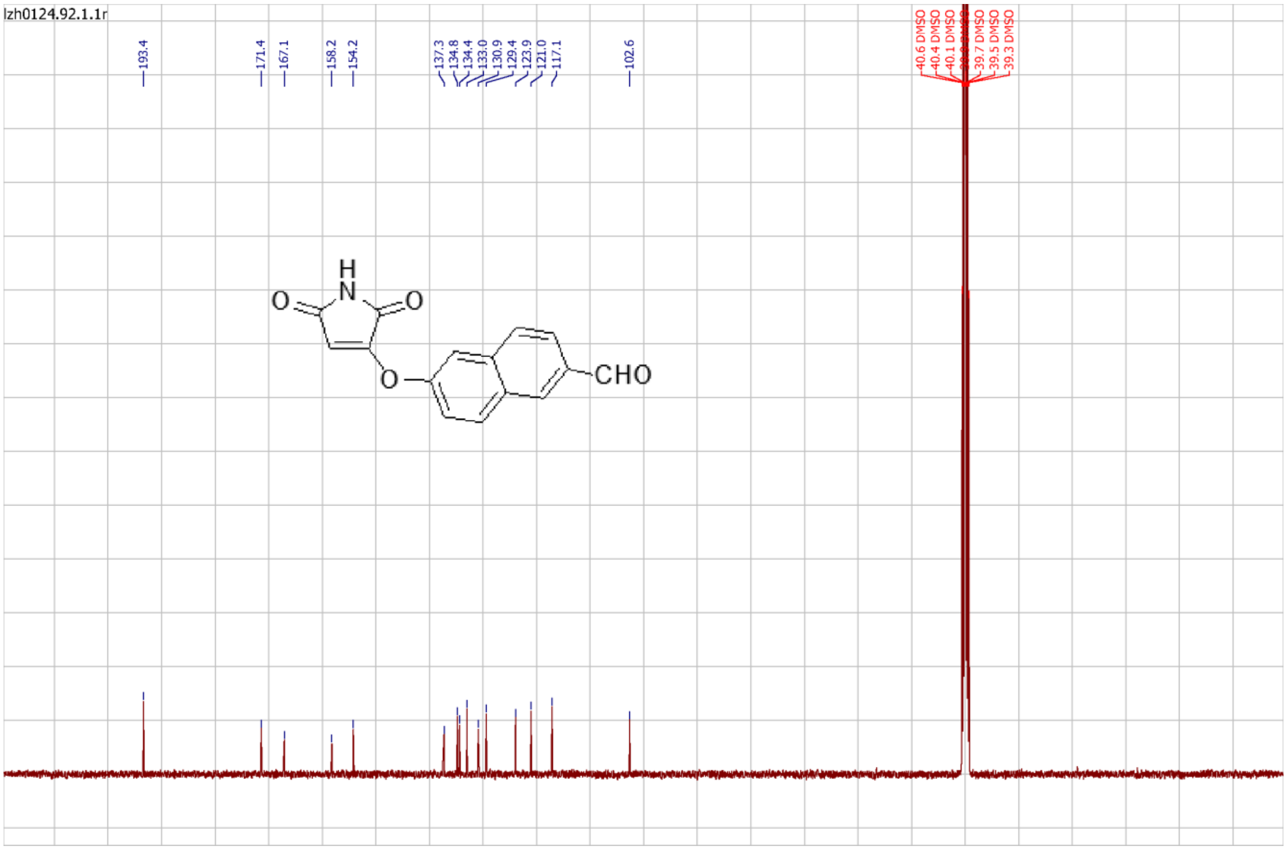

lzh0124.92.1.1r
193.4
171.4
167.1
158.2
154.2
137.3
134.8
134.4
133.0
130.9
129.4
123.9
121.0
117.1
102.6
40.6 DMSO
40.4 DMSO
40.1 DMSO
39.7 DMSO
39.5 DMSO
39.3 DMSO
H
N
O
O
O
CHO
